# Expression of delta-catenin is associated with progression of human astrocytoma

**DOI:** 10.1186/1471-2407-11-514

**Published:** 2011-12-12

**Authors:** Wang MingHao, Dong Qianze, Zhang Di, Wang YunJie

**Affiliations:** 1 Department of Neurosurgery, First Affiliated Hospital of China Medical University, Shenyang 110001, PR China; 2 Department of Pathology, First Affiliated Hospital of China Medical University, Shenyang, PR China

## Abstract

**Background:**

δ-Catenin (*CTNND2*), which encodes a scaffold protein in humans, has been found in a few malignancies. However, the expression pattern and contribution of δ-catenin to astrocytoma progression are unclear.

**Methods:**

We investigated δ-catenin expression in human astrocytoma samples and its function in astrocytoma cell lines using immunohistochemistry, siRNA knockdown, transfection, MTT, transwell migration and Rac1 pulldown techniques.

**Results:**

δ-Catenin protein expression was detected in cytoplasm of astrocytoma cells by immunohistochemistry. Analysis showed that grade I astrocytoma (0%, 0/11) and glial cells from normal brain tissue exhibited negative staining. δ-Catenin expression was significantly higher in grade III-IV (35%, 29/84) compared to grade II astrocytoma cells (18%, 11/61); *p *< 0.01). In addition, *CTNND2 *overexpression promoted proliferation, invasion and Rac1 activity of U251 astrocytoma cells. Treatment of δ-catenin-transfected cells with a Rac1 inhibitor decreased Rac1 activity and invasion. δ-Catenin knockdown in U87 glioblastoma cell decreased cell proliferation, invasion and Rac1 activity.

**Conclusion:**

The results suggest that δ-catenin expression is associated with the malignant progression of astrocytoma and promotes astrocytoma cell invasion through upregulation of Rac1 activity. δ-Catenin expression levels may serve as a useful marker of the biological behavior of astrocytoma cells.

## Background

Astrocytoma arises from neural stem or progenitor cells in the central nervous system. It is the most common primary brain tumor and accounts for approximately 60% of all brain tumors. Despite combined treatment strategies, which include surgery, radiotherapy and chemotherapy, the prognosis for high-grade astrocytoma, especially glioblastoma multiforme, has changed little over the past 10 years, with a median survival of only approximately 1 year [1]. The clinical symptoms and prognosis are closely correlated with tumor location and size and histological grade. Although the histological grade partly reflects the malignant features of astrocytoma, it cannot give an indication of the exact mechanism of tumor invasion and recurrence [2,3]. Thus, it is important to understand the molecular mechanism of astrocytoma cell invasion and identify effective markers in tumorigenesis and progression.

The expression of δ-catenin was initially found to be limited to brain neurons and it can bind to presenilin 1, which is involved in the progression of Alzheimer's disease [4-6]. δ-Catenin was also implicated in the maintenance of synaptic function and transmission of downstream signals as a scaffold protein [7-11]. It was reported that δ-catenin overexpression changed the morphology of MDCK cells, including the elaboration of lamellipodia [12]. Furthermore, δ-catenin can regulate the activity of small GTPases and therefore induces dendritic protrusions from cells [13]. Small GTPases are critical mediators of cytoskeletal reorganization, signal transduction and gene expression pathways [14,15]. As one of these GTPases, Rac1 is a pleiotropic regulator of many cellular processes, including the cell cycle, cell-cell adhesion, motility (through the actin network) and epithelial differentiation. δ-Catenin also affects invasion and metastasis of lung cancer cells via regulation of small GTPase activity [16]. These studies suggest that δ-catenin plays an important role in many human cancers. Our previous study showed that δ-catenin was overexpressed in non-small-cell lung cancer and enhanced cancer cell invasion through small GTPase regulation. However, the expression pattern of δ-catenin in glial cells and human astrocytoma cells is not clear; the relationship between its expression and clinicopathological factors and biological behavior still needs to be addressed.

In the present study, we examined the expression of δ-catenin in 156 astrocytoma tissue specimens and analyzed the correlation between its expression and clinicopathological factors. We also investigated the effect of δ-catenin on Rac1 activity and the biological behavior of astrocytoma cell lines.

## Methods

### Patients and specimens

The study protocol was approved by the institutional review board of China Medical University. Primary tumor specimens and adjacent normal brain tissue were obtained from 156 patients diagnosed with astrocytoma who underwent resection in the First Affiliated Hospital of China Medical University between 2005 and 2008. None of the patients had received radiotherapy or chemotherapy before surgical resection. There were 95 male (60.9%) and 61 female (39.1%) patients with a median age of 43 years (range 15-67 years). The histological diagnosis and differentiation grade were evaluated for sections stained with hematoxylin and eosin according to the World Health Organization (WHO) classification guidelines. All 156 specimens were re-evaluated with respect to histological subtype and tumor grade. Tumors were graded and classified into grade I (11), grade II (61), grade III (51), and grade IV (33) according to WHO guidelines (2007).

### Cell culture and transfection

U251 and U87 cell lines were obtained from American Type Culture Collection (Manassas, VA, USA). The cells were cultured in DMEM (Invitrogen, Carlsbad, CA, USA) containing 10% fetal calf serum (Invitrogen), 100 IU/ml penicillin (Sigma, St. Louis, MO, USA), and 100 μg/ml streptomycin (Sigma). Cells were grown on sterilized culture dishes and were passaged every 2 days with 0.25% trypsin (Invitrogen). The pCMV5-FLAG/δ-catenin plasmid was a kind gift from Dr. Shun-ichi Nakamura at Kobe University and was transfected into cells using Attractene Transfection (Qiagen, Hamburg, Germany). A pCMV5 empty vector was used as a negative control. DharmaFECT1 reagent was used for siRNA transfection (Qiagen, Chicago, IL, USA) according to the manufacturer's instructions. The protein level was assessed 48 h later by western blotting. The δ-catenin siRNA sequences were 5'-CUA CGU UGA CUU CUA CUC AUU-3' and 5'-UGA GUA GAA GUC AAC GUA GUU-3' and were transfected into U87 cell lines, which express relatively high levels of δ-catenin. Nonsilencing siRNA sequences were used as a negative control (5'-UUC UCC GAA CUU GUC ACA UUU-3' and 5'-AUG UGA CAA GUU CGG AGA AUU-3').

### Immunohistochemistry

Surgically excised tumor specimens were fixed with 10% neutral formalin and embedded in paraffin, and 4-μm-thick sections were prepared. Normal brain neurons present in the tumor slides was used as positive control. Immunostaining was performed using the avidin-biotin-peroxidase complex method (Ultrasensitive™, MaiXin, Fuzhou, China). The sections were deparaffinized in xylene, rehydrated with graded alcohol, and then boiled in 0.01 M citrate buffer (pH 6.0) for 2 min in an autoclave. Hydrogen peroxide (0.3%) was applied to block endogenous peroxide activity and the sections were incubated with normal goat serum to reduce nonspecific binding. Tissue sections were incubated with δ-catenin mouse monoclonal antibody (1:100 dilution; Santa Cruz Biotechnology, Santa Cruz, CA, USA). Mouse immunoglobulin (at the same concentration as for the antigen-specific antibody) was used as a negative control. Staining for both antibodies was performed at room temperature for 2 h. Biotinylated goat antimouse serum IgG was used as a secondary antibody. After washing, the sections were incubated with streptavidin-biotin conjugated with horseradish peroxidase, and the peroxidase reaction was developed with 3,3'-diaminobenzidine tetrahydrochloride. Counterstaining with hematoxylin was performed and the sections were dehydrated in ethanol before mounting.

Two independent blinded investigators examined all tumor slides randomly. Five views were examined per slide, and 100 cells were observed per view at 400 × magnification. Immunostaining of δ-catenin was scored on a semiquantitative scale by evaluating in representative tumor areas the intensity and percentage of cells showing significantly higher immunostaining than control cells in normal brain tissues. Cytoplasmic immunostaining in tumor cells was considered positive staining. We counted 400 tumor cells and calculated the percentage of positively stained cells. The proportion of cells exhibiting δ-catenin expression was categorized as follows: 0, absent; 1, 1-25%; 2, 26-50%; 3, 51-75%; and 4, ≥ 76%. The staining intensity was categorized as follows: 0, negative; 1, moderate; and 2, strong. The proportion and intensity scores were then multiplied to obtain a total score. To obtain final statistical results, a score < 2 was considered negative and scores ≥ 2 were considered positive.

### Western blot analysis

Total proteins from tissue and cells were extracted in lysis buffer (Pierce, Rockford, IL) and quantified using the Bradford method. Samples of 50 μg of protein were separated by SDS-PAGE. Samples were transferred to polyvinylidene fluoride membranes (Millipore, Billerica, MA, USA) and incubated overnight at 4°C with antibody against δ-catenin (1:1000; Santa Cruz) and a mouse monoclonal antibody against β-actin (1:500; Santa Cruz). After incubation with peroxidase-coupled antimouse IgG (Santa Cruz) at 37°C for 2 h, bound proteins were visualized using ECL (Pierce) and detected using a BioImaging System (UVP Inc., Upland, CA, USA). Relative protein levels were quantified using β-actin as a loading control.

### Colony formation and MTT assays

U251 and U87 cells were transfected with siRNA or a δ-catenin plasmid for 48 h and then plated into three 6-cm cell culture dishes (1000 per dish) and incubated for 12 days. Plates were washed with PBS and stained with Giemsa. The number of colonies with more than 50 cells was counted. The colonies were manually counted using a microscope.

Cells were plated in 96-well plates in medium containing 10% FBS at approximately 3000 cells per well 24 h after transfection. For quantitation of cell viability, cultures were stained after 4 days in MTT assays. In brief, 20 μl of 5 mg/ml MTT (thiazolyl blue) solution was added to each well and incubated for 4 h at 37°C. The medium was removed from each well and the resulting MTT formazan was solubilized in 150 μl of DMSO. Each solution was measured spectrophotometrically at 490 nm.

### Matrigel invasion assay

Cell invasion assays were performed in 24-well Transwell chambers with a pore size of 8 μm and the inserts were coated with 20 μl of Matrigel (1:3 dilution, BD Bioscience, San Diego, CA, USA). At 48 h after transfection, cells were trypsinized and transferred to the upper Matrigel chamber in 100 μl of serum-free medium containing 3 × 10^5 ^cells and incubated for 16 h. Medium supplemented with 10% FBS was added to the lower chamber as the chemoattractant. Then non-invading cells on the upper membrane surface were removed with a cotton tip and cells that had passed through the filter were fixed with 4% paraformaldehyde and stained with hematoxylin. The number of invaded cells were counted in 10 randomly selected high-power fields under a microscope. The experiments were performed in triplicate.

### Rac1 pulldown assayM

Rac1 activity was measured using a Active Rac1 Pull-Down and Detection Kit (Pierce). The Rac1 inhibitor NSC23766 was from Calbiochem (Merck, Whitehouse Station, NJ, USA). Cells were treated for 6 h at a concentration of 50 μM.

### Statistical analysis

SPSS version 11.5 for Windows was used for all statistical analyses. A χ^2 ^test was used to examine possible correlations between δ-catenin expression and clinicopathologic factors. Student's t-test was used to compare densitometry data on focus numbers between control and δ-catenin-transfected cells. All p values are based on a two-sided statistical analysis, and *p *< 0.05 was considered to indicate statistical significance.

## Results

### Expression and localization of δ-catenin in normal brain tissue and astrocytoma cells

**Figure 1:**
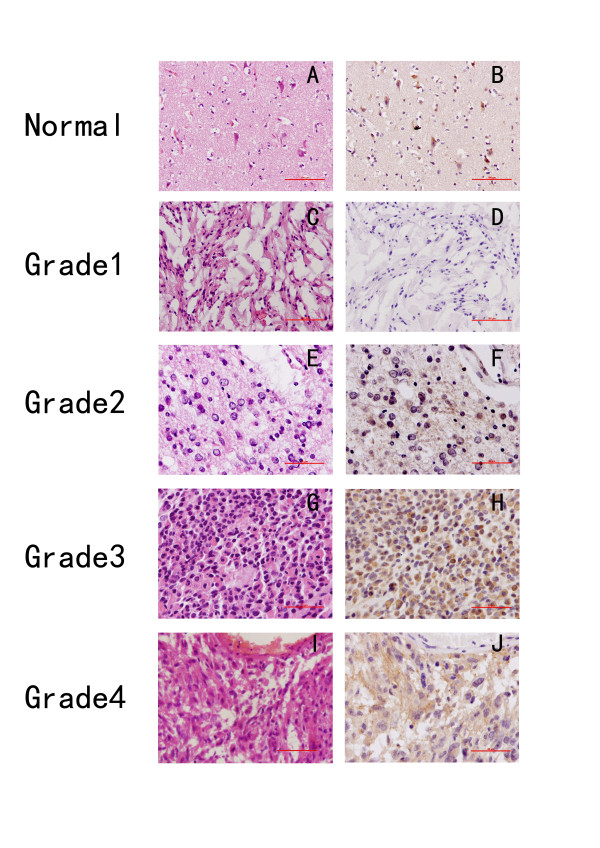
**Expression of δ-catenin in resected astrocytoma tissue**. (A) HE staining of normal brain tissue. (B) Immunohistochemical staining of δ-catenin protein in normal brain tissue was negative in glial cells and positive in neurons. (C) HE staining of pilocytic astrocytoma (grade I). (D) Negative δ-catenin staining in pilocytic astrocytoma (grade I). (E) HE staining of grade II astrocytoma. (F) Positive δ-catenin staining (weak staining) in the cytoplasm of grade II glioma. (G) HE staining of grade III astrocytoma. (H) Strong cytoplasmic δ-catenin staining in grade III astrocytoma. (I) HE staining of glioblastoma (grade IV). (J) Strong cytoplasmic δ-catenin staining in glioblastoma (grade IV).

In cells from normal brain tissue, only neurons showed strong cytoplasmic (include axon) expression of δ-catenin, whereas glial cells showed no expression (Figure 1a, b). Cytoplasmic staining was present in a subset of astrocytoma cases, while no staining was detected in sections from the same samples subjected to immunohistochemical analysis using mouse immunoglobulin. Of the 156 patients in the study, 40 (25.6%) showed positive δ-catenin expression. δ-Catenin expression predominantly occurs in the cytoplasmic compartment of tumor cells. We analyzed the correlation between δ-catenin protein expression and the histological staging of astrocytoma. As shown in Table 1 Grade I astrocytoma (0%, 0/11) and normal glial cells exhibited negative staining (Figure 1c, d). In grade III-IV astrocytoma (35%, 29/84; Figure 1g-j), δ-catenin expression was significantly higher than in grade II astrocytoma (18%, 11/61; Figure 1e, f, p < 0.01), which suggests that δ-catenin expression is associated with astrocytoma progression.

**Table 1:** The relationship between δ-catenin and clinicopathological features

Clinical parameters	Number	δ-catenin		p value
		
		Negative	Positive	
Age				

< 43	78	61	17	0.359

≥ 43	78	55	23	

Gender				

Male	95	69	26	0.578

Female	61	47	14	

Grading				

Grade I	11	11	0	0.009*

Grade II	61	51	11	

High Grade (III-IV)	84	55	29	

* *p *< 0.05

We examined the difference between the expression pattern of δ-catenin according to tumor location. As shown in Table 2 the positive rate of δ-cateninin supratentorial astrocytomas was 29.3%, much higher than the rate in infratentorial astrocytomas (13.3%). (*p *< 0.05).

**Table 2:** Association of δ-catenin expression with tumor location

		δ-catenin	
**Sites**	**Total**	**Negative**	**Positive**

Supratentorial			

Hemisphere	124	87(70.2%)	37(29.8%)

Diencephalon	2	2(100%)	0(0%)

Total	126	89(70.7)	37(29.3%)

Infratentorial			

Cerebellum	17	13(76.5%)	4(23.5%)

Brain stem	5	5(100%)	0(0%)

Spinal cord	8	8(100%)	0(0%)

Total	30	26(86.7%)	4(13.3%)*

* Supratentorial versus Infratentorial, *p *< 0.05

### δ-Catenin overexpression promotes astrocytoma cell proliferation and invasion via Rac1

To determine whether δ-catenin enhances the proliferation and invasion of astrocytoma cells, we carried out δ-catenin transfection in the U251 astrocytoma cell line. siRNA knockdown was performed in the U87 glioblastoma cell line.

**Figure 2:**
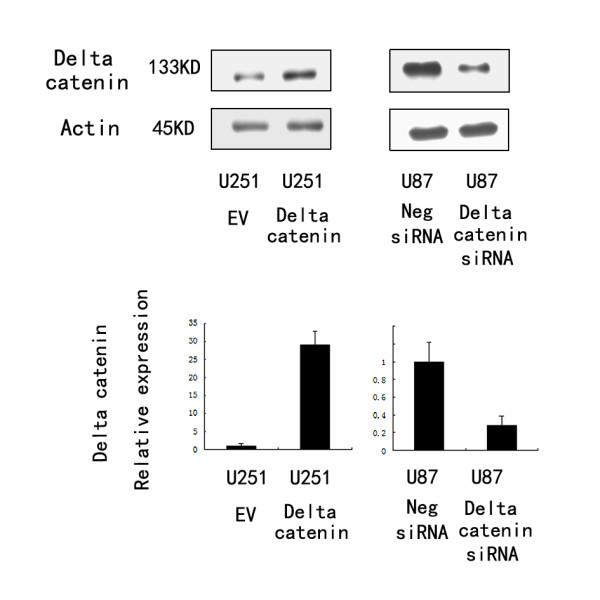
**δ-Catenin transfection of U251 cells and siRNA knockdown in U87 cells**. Western blot and real-time PCR analyses showed that U251 cells transfected with the δ-catenin plasmid expressed higher levels of δ-catenin. δ-Catenin expression in U87 cells transfected with siRNA was downregulated.

**Figure 3:**
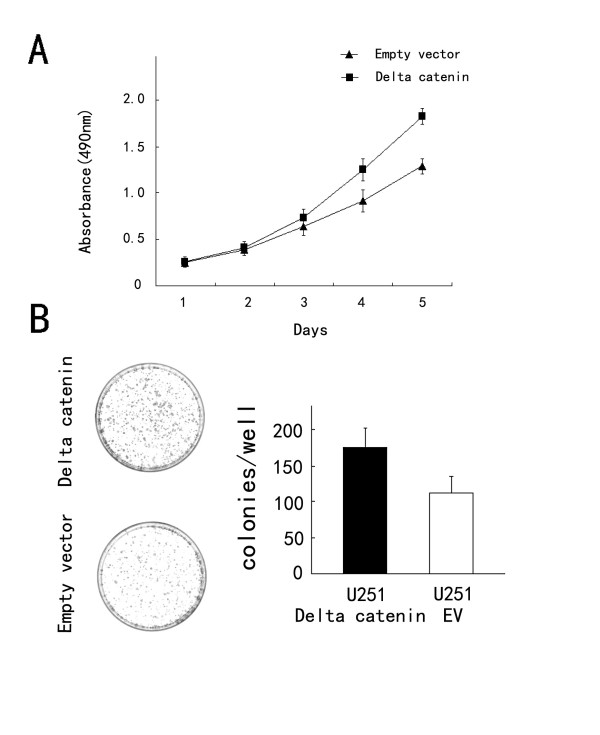
**δ-Catenin transfection promotes cancer cell proliferation in U251 cells**. (A) MTT assays were performed after δ-catenin transfection. An increase in absorbance was observed. (B) Assessment of the clonogenic potential of δ-catenin-transfected glioma cells. The number of colonies formed by cells treated with δ-catenin was greater than for control cells (*p *< 0.05). Columns, mean; bars, SD.

**Figure 4:**
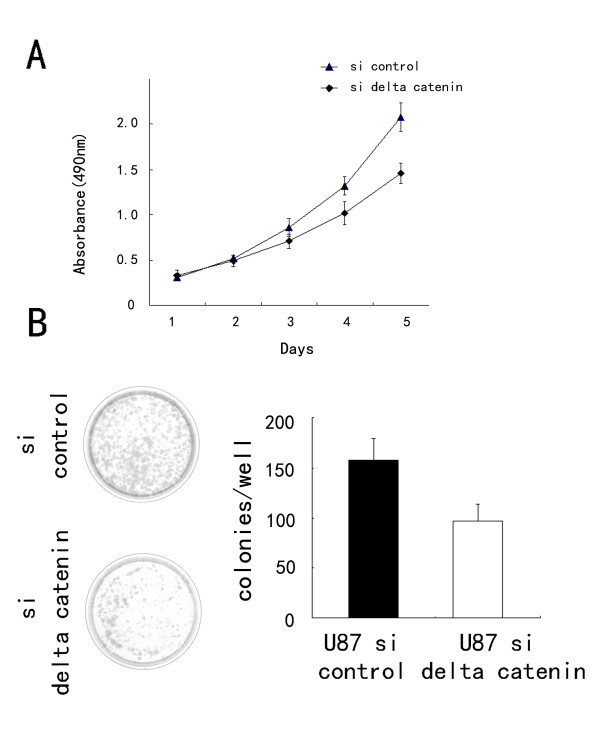
**δ-Catenin knockdown inhibits proliferation in U87 cells**. (A) MTT assays were performed after δ-catenin siRNA transfection. A decrease in absorbance was observed. (B) Assessment of the clonogenic potential of δ-catenin-transfected glioma cells. The number of colonies formed by cells treated with δ-catenin siRNA was less than for control cells (*p *< 0.05). Columns, mean; bars, SD.

**Figure 5:**
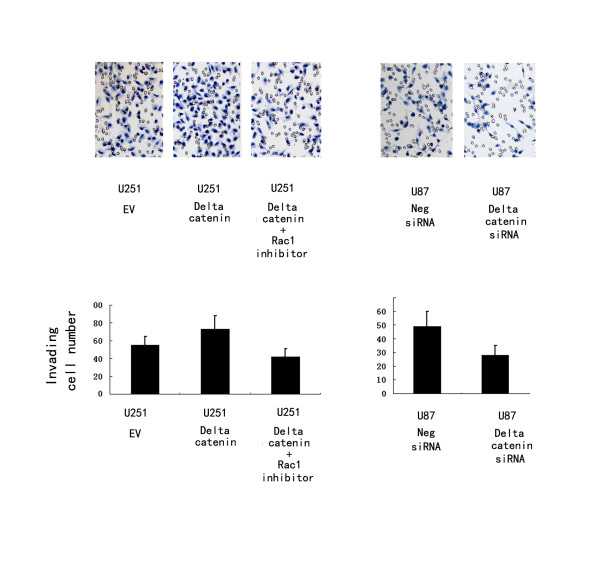
**Invasion assay of cells transfected with δ-catenin and siRNA**. δ-Catenin transfection promoted cell invasion in U251 cells, while siRNA knockdown of δ-catenin had an inhibitory effect. The numbers of cells invading onto the lower surface of the filter was significantly different (*p *< 0.05). Columns, mean; bars, SD.

**Figure 6:**
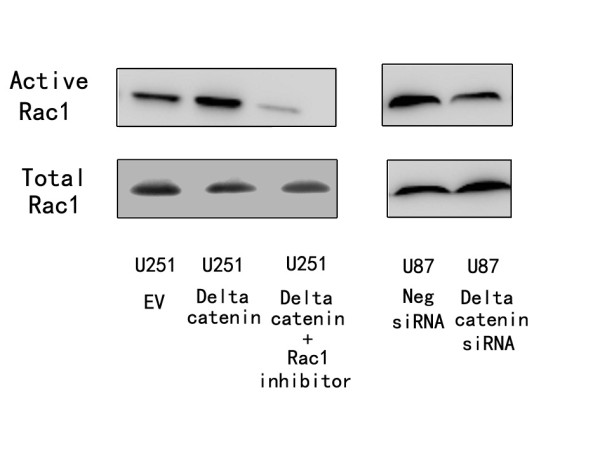
**δ-Catenin transfection and siRNA knockdown affect Rac1 activity**. Overexpression of the δ-catenin gene promoted Rac1 activity in U251 astrocytoma cells. siRNA treatment and a Rac1 inhibitor decreased this activity.

As shown in Figure 2, δ-catenin transfection considerably increased protein levels at 48 h after treatment, whereas δ-catenin siRNA knockdown decreased δ-catenin protein in U87 cells. The proliferation rate was determined by MTT assay. A significant increase in proliferation was observed in U251 cells overexpressing δ-catenin compared to cells transfected with the empty vector (Figure 3a). Consistent with the MTT results, colony formation assays showed that transfection of δ-catenin in U251 cells led to an increase in focus numbers (empty vector 109 ± 23 vs δ-catenin 174 ± 26, *p *< 0.05; Figure 3b). δ-Catenin also promoted tumor cell invasion in a Matrigel assay (empty vector 55 ± 10 vs δ-catenin 73 ± 15, *p *< 0.05; Figure 4a). In U251 cells overexpressing δ-catenin, immunoblotting showed total Rac1 protein levels were unchanged, but a pulldown assay revealed that Rac1 activity increased significantly (Figure 4b). Conversely, δ-catenin knockdown decreased the proliferation, colony formation ability (control158 ± 21 vs siRNA δ-catenin 97 ± 17, *p *< 0.05; Figure 5) and invasiveness of U87 cells (control 59 ± 7 vs siRNA δ-catenin 36 ± 5, *p *< 0.05). δ-Catenin knockdown also decreased Rac1 activity (Figure 6).

To explore whether the effect of δ-catenin on cell invasion was dependent on Rac1 activity, we measured cell invasion and Rac1 activity after treatment of δ-catenin-transfected cells with the Rac1 inhibitor NSC23766. The inhibitor abolished the effect of δ-catenin on cell invasion (δ-catenin 73 ± 15 vs δ-catenin + Rac1 inhibitor 42 ± 9, *p *< 0.05).

## Discussion

δ-catenin plays an important role in dendritic morphogenesis, which is associated with changes in the activity of small GTPases [7,13]. The formation and number of dendritic protrusions are indicative of the malignant phenotype of cells, so it is possible that δ-catenin promotes a malignant phenotype in tumor cells. It has been reported that δ-catenin expression was increased in over 80% (55/65) of prostatic adenocarcinomas, and was associated with higher Gleason scores [4]. Some researchers also observed gene amplification of δ-catenin in cervical and bladder carcinoma. δ-catenin mRNA is probably present in esophagus, ovary and breast tumors [17,18]. Our previous study showed that δ-catenin was overexpressed in non-small-cell lung cancer and was correlated with clinicopathological factors [16]. However, the expression pattern and clinical significance of δ-catenin are poorly defined in other human cancers such as astrocytoma.

Analysis of mRNA in primary cultures of neurons and glial cells prepared from fetal rat brain showed that δ-catenin expression levels were higher in neurons than in glial cells [19]. However, its protein expression in neurons and glial cells has not been investigated. In this study, we found strong cytoplasmic δ-catenin expression in neurons but negative staining in human glial cells such as astrocytes, which may due to the low mRNA copy numbers of δ-catenin. Although there was no expression of δ-catenin in 11 grade I astrocytoma cases, positive δ-catenin was found in 18% of grade II and 35% of grade III-IV astrocytoma cases, which suggests that δ-catenin is positively associated with higher grade astrocytoma. Positive rate of δ-catenin in low grade astrocytoma is very low, especially in grade I astrocytoma (Negative expression). and its utilization in the distinguishing grade I astrocytoma from reactive gliosis was limited. The low grade astrocytoma with positive δ-catenin expression may transform into high grade astrocytoma. These findings indicate that δ-catenin might play an important role in astrocytoma progression and could serve as a potential biomarker for the biological behavior of astrocytoma cells.

To explore the relationship between δ-catenin and the biological behavior of astrocytoma cells, we transfected a δ-catenin plasmid into the U251 cell line, which has low δ-catenin expression, and knocked down δ-catenin in the U87MG cell line, which has high δ-catenin expression. We found that δ-catenin overexpression caused a significant increase in the proliferation rate, colony formation and invasion ability of U251 cell lines, with a significant increase in Rac1 activity, which was in accordance with our previous results for lung cancer [16]. On the contrary, δ-catenin knockdown decreased the proliferation rate, colony formation ability and invasiveness of U87 cells, with a decrease in Rac1 activity. We then observed a decrease in invasiveness and Rac1 activity after treatment of δ-catenin-transfected cells with a Rac1 inhibitor, which demonstrates that δ-catenin regulates cell invasion in a Rac1-dependent manner. These results suggest that δ-catenin might enhance the invasive ability of astrocytoma cells via Rac1. However, the exact mechanism of Rac1 regulation by δ-catenin is not clear. In addition, previous studies proved that δ-catenin has an influence in other small GTPases, such as Cdc42 and RhoA, which was not investigated in glioma cell lines [13]. Further studies are required to address these issues.

## Conclusion

In conclusion, overexpression of δ-catenin in astrocytoma cells was correlated with higher grade and δ-catenin was identified as an important oncoprotein in maintaining the malignant behavior of glioma cells. Overexpression of δ-catenin activated Rac1 and promoted astrocytoma cell proliferation and invasion. Thus, δ-catenin is a candidate target protein for future astrocytoma therapeutics.

## Competing interests

The authors declare that they have no competing interests.

## Authors' contributions

WM and ZD carried out the immunohistochemistry, cell biological analysis and immunoassay. DQ participated in the design of the study and drafted the manuscript. WY participated in its design. All authors read and approved the final manuscript.

## Pre-publication history

The pre-publication history for this paper can be accessed here:

http://www.biomedcentral.com/1471-2407/11/514/prepub

## References

[B1] Bondy ML Scheurer ME Malmer B Barnholtz-Sloan JS Davis FG Il'yasova D Kruchko C McCarthy BJ Rajaraman P Schwartzbaum JA Sadetzki S Schlehofer B Tihan T Wiemels JL Wrensch M Buffler PA Brain tumor epidemiology: consensus from the Brain Tumor Epidemiology Consortium Cancer 2008 113 7 Suppl 1953 1968 18798534 10.1002/cncr.23741 PMC2861559

[B2] Walker PR Calzascia T de Tribolet N Dietrich PY T-cell immune responses in the brain and their relevance for cerebral malignancies Brain Res Brain Res Rev 2003 42 2 97 122 12738053 10.1016/s0165-0173(03)00141-3

[B3] van den Bent MJ Hegi ME Stupp R Recent developments in the use of chemotherapy in brain tumours Eur J Cancer 2006 42 5 82 588 10.1016/j.ejca.2005.06.031 16427778

[B4] Lu Q Dobbs LJ Gregory CW Lanford GW Revelo MP Shappell S Chen YH Increased expression of delta-catenin/neural plakophilin-related armadillo protein is associated with the down-regulation and redistribution of E-cadherin and p120ctn in human prostate cancer Hum Pathol 2005 36 10 1037 1048 10.1016/j.humpath.2005.07.012 16226102

[B5] Tanahashi H Tabira T Isolation of human delta-catenin and its binding specificity with presenilin 1 Neuroreport 1999 10 3 563 568 10.1097/00001756-199902250-00022 10208590

[B6] Zhou J Liyanage U Medina M Ho C Simmons AD Lovett M Kosik KS Presenilin 1 interaction in the brain with a novel member of the Armadillo family Neuroreport 1997 8 8 2085 2090 10.1097/00001756-199705260-00054 9223106

[B7] Kim H Han JR Park J Oh M James SE Chang S Lu Q Lee KY Ki H Song WJ Kim K Delta-catenin-induced dendritic morphogenesis. An essential role of p190RhoGEF interaction through Akt1-mediated phosphorylation J Biol Chem 2008 283 2 977 987 17993462 10.1074/jbc.M707158200 PMC2265781

[B8] Lu Q Mukhopadhyay NK Griffin JD Paredes M Medina M Kosik KS Brain armadillo protein delta-catenin interacts with Abl tyrosine kinase and modulates cellular morphogenesis in response to growth factors J Neurosci Res 2002 67 5 618 624 10.1002/jnr.10151 11891774

[B9] Ochiishi T Futai K Okamoto K Kameyama K Kosik KS Regulation of AMPA receptor trafficking by delta-catenin Mol Cell Neurosci 2008 39 4 499 507 10.1016/j.mcn.2008.06.002 18602475

[B10] Arikkath J Reichardt LF Cadherins and catenins at synapses: roles in synaptogenesis and synaptic plasticity Trends Neurosci 2008 31 9 487 494 10.1016/j.tins.2008.07.001 18684518 PMC2623250

[B11] Kim K Sirota A Chen Yh YH Jones SB Dudek R Lanford GW Thakore C Lu Q Dendrite-like process formation and cytoskeletal remodeling regulated by delta-catenin expression Exp Cell Res 2002 275 2 171 184 10.1006/excr.2002.5503 11969288

[B12] Lu Q Paredes M Medina M Zhou J Cavallo R Peifer M Orecchio L Kosik KS delta-catenin, an adhesive junction-associated protein which promotes cell scattering J Cell Biol 1999 144 3 519 532 10.1083/jcb.144.3.519 9971746 PMC2132907

[B13] Abu-Elneel K Ochiishi T Medina M Remedi M Gastaldi L Caceres A Kosik KS A delta-catenin signaling pathway leading to dendritic protrusions J Biol Chem 2008 283 47 32781 91 10.1074/jbc.M804688200 18809680

[B14] Vega FM Ridley AJ SnapShot Rho family GTPases Cell 2007 129 7 1430 17604728 10.1016/j.cell.2007.06.021

[B15] Sahai E Marshall CJ RHO-GTPases and cancer Nat Rev Cancer 2002 2 2 133 142 10.1038/nrc725 12635176

[B16] Zhang JY Wang Y Zhang D Yang ZQ Dong XJ Jiang GY Zhang PX Dai SD Dong QZ Han Y Zhang S Cui QZ Wang EH delta-Catenin promotes malignant phenotype of non-small cell lung cancer by non-competitive binding to E-cadherin with p120ctn in cytoplasm J Pathol 222 1 76 88 10.1002/path.2742 20593408

[B17] Zheng M Simon R Mirlacher M Maurer R Gasser T Forster T Diener PA Mihatsch MJ Sauter G Schraml P TRIO amplification and abundant mRNA expression is associated with invasive tumor growth and rapid tumor cell proliferation in urinary bladder cancer Am J Pathol 2004 165 1 63 69 10.1016/S0002-9440(10)63275-0 15215162 PMC1618551

[B18] Huang FY Chiu PM Tam KF Kwok YK Lau ET Tang MH Ng TY Liu VW Cheung AN Ngan HY Semi-quantitative fluorescent PCR analysis identifies PRKAA1 on chromosome 5 as a potential candidate cancer gene of cervical cancer Gynecol Oncol 2006 103 1 219 225 10.1016/j.ygyno.2006.02.028 16595147

[B19] Kawamura Y Fan QW Hayashi H Michikawa M Yanagisawa K Komano H Expression of the mRNA for two isoforms of neural plakophilin-related arm-repeat protein/delta-catenin in rodent neurons and glial cells Neurosci Lett 1999 277 3 185 188 10.1016/S0304-3940(99)00875-7 10626844

